# DNA barcodes evidence the contact zone of eastern and western caddisfly lineages in the Western Carpathians

**DOI:** 10.1038/s41598-021-03411-8

**Published:** 2021-12-15

**Authors:** Jana Bozáňová, Fedor Čiampor, Tomasz Mamos, Michal Grabowski, Zuzana Čiamporová-Zat’ovičová

**Affiliations:** 1 ZooLab, Plant Science and Biodiversity Centre, Slovak Academy of Sciences, Dúbravská cesta 9, 845 23 Bratislava, Slovak Republic; 2 Department of Ecology, Faculty of Natural Sciences, Comenius University in Bratislava, Ilkovičova 6, 842 15 Bratislava, Slovak Republic; 3 Department of Invertebrate Zoology and Hydrobiology, Faculty of Biology & Environmental Protection, University of Lodz, Banacha 12/16, 90-237 Lodz, Poland

**Keywords:** Haplotypes, Population genetics, Ecology, Ecology, Limnology

## Abstract

The region of the Western Carpathians is, among other aspects, very important for survival and diversity of European freshwater fauna due to the presence of a large number of (sub)mountain springs and streams. However, these ecologically and faunistically diversified habitats are still understudied in the context of genetic diversity and population structure of their inhabitants. This study focuses on genetic diversity and distribution patterns of the caddisfly *Rhyacophila tristis*, common and widespread representative of mountain freshwater fauna. Analysis of the COI mitochondrial marker revealed presence of the western and eastern lineages, with samples from both lineages being grouped in BOLD (Barcode of Life Data System) into separate BINs (Barcode Index Numbers). Our data indicates that eastern lineage (BIN_E) is more closely related to the Balkan populations than to co-occurring western lineage (BIN_W), and that the contact zone of the lineages passes through the W Carpathians. The study revealed phylogeographic and demographic differences between lineages, supporting hypothesis of their evolutionary independence and specific ecological preferences. The obtained genetic data of the *R. tristis* population from W Carpathians improved our knowledge about population genetics of this aquatic species and can contribute to understanding the state and evolution of biodiversity of freshwater ecosystems in Europe.

## Introduction

Population genetic studies are inevitable for understanding the mechanisms of distribution and dispersal of species under the influence of past climate changes. The acquired results may subsequently help to estimate evolution of species affected by current climate change, as they provide valuable information on the size of the gene pool or the degree of species adaptation in relation to environmental changes^1–9^.

**Figure 1:**
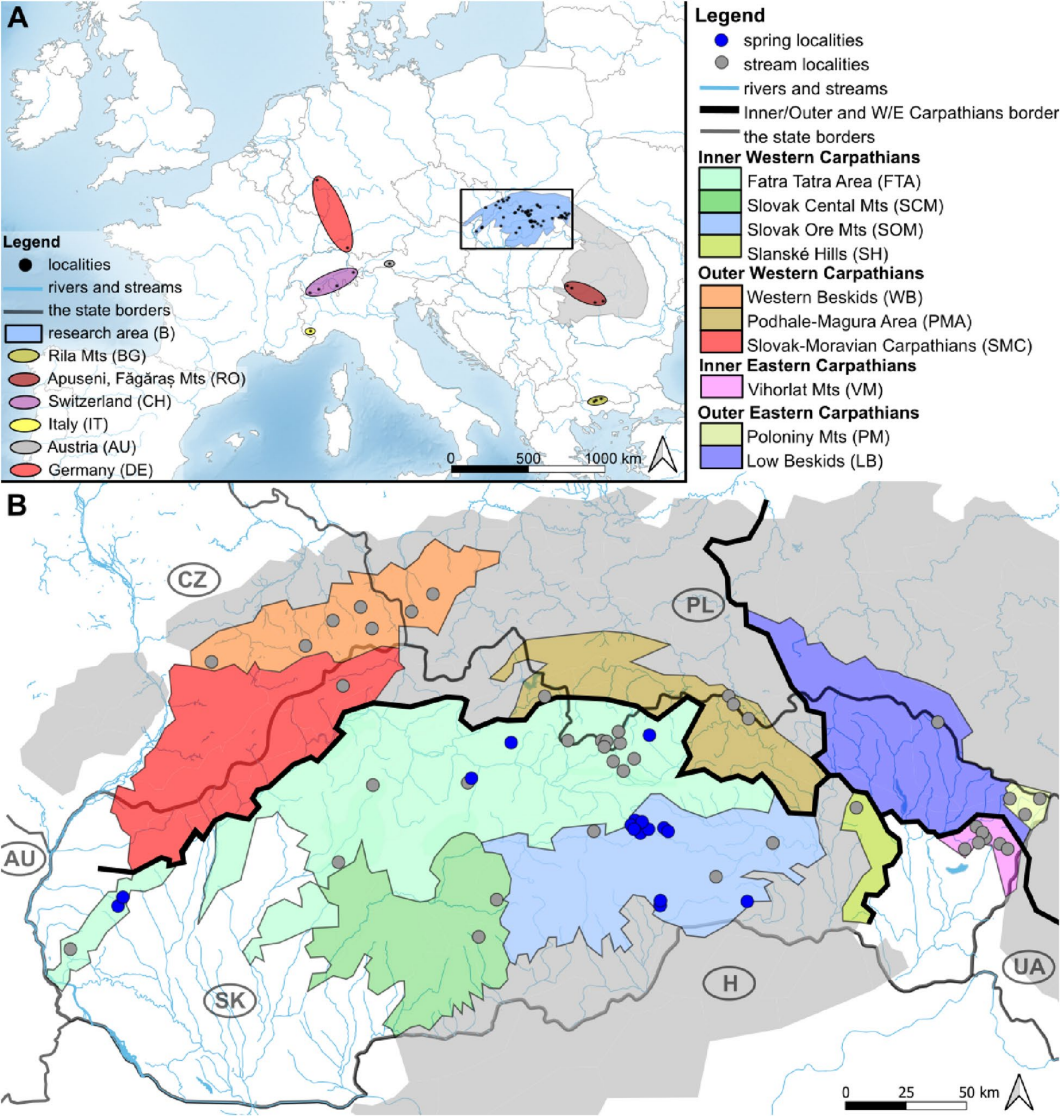
Distribution of the *R. tristis* sites. (**A**) Map of the research area (rectangle) within Europe and 15 localities from outside of the research area (ellipses); (**B**) Research area with 58 sampling sites (15 springs, 43 streams) divided into ten physiographic units represented by different fill colours (see legend). Abbreviations: Slovakia (SK), Hungary (H), Ukraine (UA), Poland (PL), Czech Republic (CZ), Austria (AU), Bulgaria (BG), Romania (RO). Map created with QGIS 3.16 (https://qgis.org/).

Recent molecular data on aquatic and terrestrial taxa show that the Western Carpathians (further W Carpathians, Fig. 1) constitute an important European biodiversity hotspot^10–16^. Some studies also document the major role of the W Carpathians as a glacial refugia for various species or genetic lineages^5,14,17–22^. The W Carpathians form a huge reservoir of freshwater, including a rich system of submountain and mountain springs and streams, which are still understudied in the context of the genetic diversity and population structure of their inhabitants. However, those few studies dealing with populations of aquatic species in the W Carpathian mountains have confirmed significantly different patterns of their genetic diversity than in the rest of Europe^23–25^.

The knowledge on the spatial distribution of genetic diversity at a species level is an essential prerequisite for understanding the resilience of biota in the changing environment across geological periods^26,27^. This is especially important for species inhabiting specific biotopes such as mountain aquatic ecosystems. The caddisfly *Rhyacophila tristis* Pictet, 1834 is one of the three species from the *R. tristis* group, widespread in Europe. Adults of *R. tristis* are relatively good fliers, which guarantees efficient dispersal. They prefer upper parts of streams, because larvae require well-oxygenated water with low levels of organic matter^28^. Bálint et al. in 2011^29^ provided the first data on the large-scale population genetics of this (sub)montane species on the European level. The study revealed strong genetic differences between populations from the western and eastern part of Europe, to which, among other factors, large distances between sites as well as sparse sampling may have contributed. It was suggested that *R. tristis* survived in an independent Pleistocene refugia both in the Alps and the Carpathians. Unfortunately, the area of the W Carpathians was not covered in this study, so the data from an important part of the species area of distribution was missing.

W Carpathians represent the northernmost part of the Alpine-Carpathian mountain chain^30^. Due to high habitat diversity of this area, including numerous montane springs and streams, we hypothesize that the genetic patterns and level of the genetic diversity found by Bálint et al. in 2011^29^ at the large geographic scale could be also detected at a much smaller area of the W Carpathians. It could also be expected that this mountain range may include a contact zone between the Alpine (western) and Carpathian (eastern) lineages of the *R. tristis*. To test these assumptions, we generated and analysed a large dataset of *R. tristis* samples from the springs and streams in the W Carpathians and adjacent areas, which also allowed us to fill the gap in the phylogeographic puzzle of this species in the European mountains.

## Results

**Figure 2:**
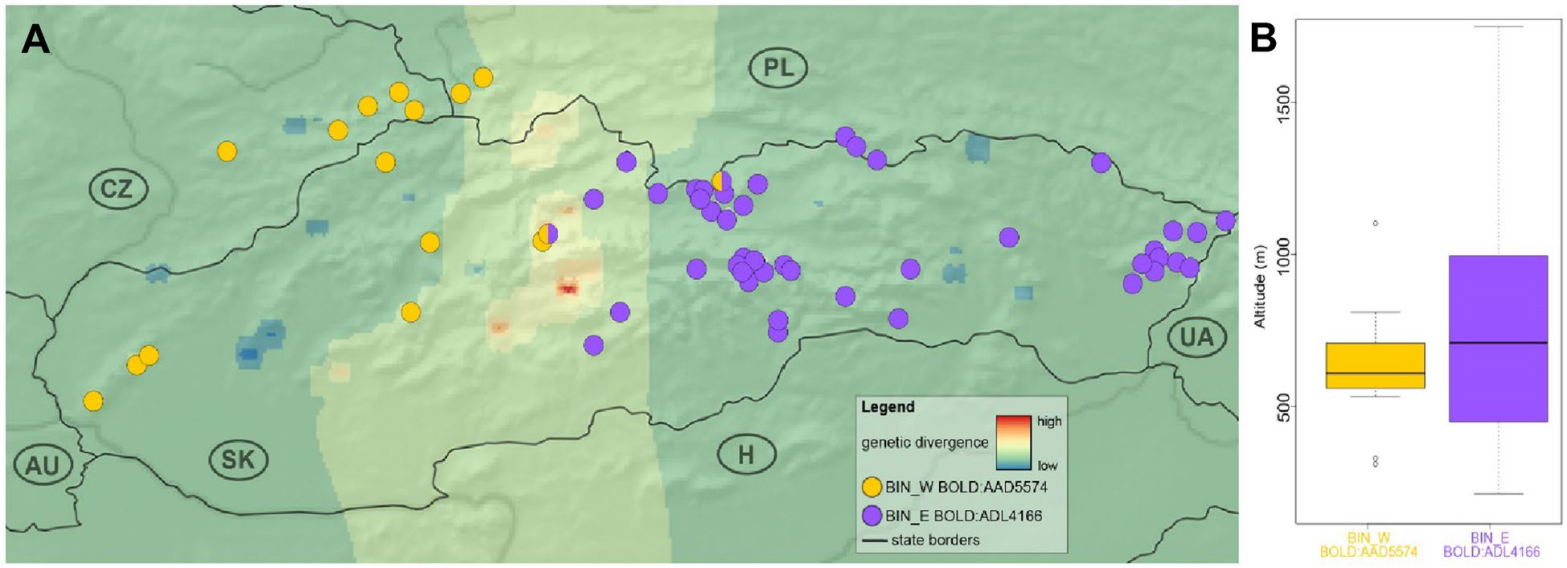
The distribution of the *R. tristis* BINs, their genetic divergence and elevation range within the studied area. (**A**) The genetic landscape map of the studied area based on 5′ COI mtDNA haplotypes with the distribution of two different BINs (BIN_W—yellow, BIN_E—violet). Abbreviations: Slovakia (SK), Hungary (H), Ukraine (UA), Poland (PL), Czech Republic (CZ) and Austria (AU). The map generated in QGIS 3.16 (https://qgis.org/); (**B**) The elevation range of both BINs. The boxplots show the distribution of the elevation above sea level for BIN_W and BIN_E. The boxes represent the interquartile distances (IQD), while the central lines through each box show the medians. The dots indicate outliers and the whiskers extend to the extreme values of the data, calculated as ± 1.5 × IQD from the median. Wilcoxon signed-rank test supported the significant differences in elevation range between two BINs (p-value < 0.05).

The caddisfly *R. tristis* had a relatively wide distribution in the studied area, both in streams and springs, although not in high numbers. The BIN (Barcode Index Number) algorithm implemented in BOLD (Barcode of Life Data System) and ASAP (Assemble Species by Automatic Partitioning) analysis resulted in identical partitioning of the 5′ COI dataset into two genetic lineages, whose distribution was largely disjunct (Fig. 2A).

The western lineage, equal to BIN *BOLD:AAD5574* (BIN_W), was recorded at 16 localities in the west, while the eastern lineage, equal to BIN *BOLD:ADL4166* (BIN_E), was found at 44 localities in the east. Both lineages (BINs) were found in sympatry only at two localities in the Fatra-Tatra area: the Biela Voda stream (T180) and Jazierce spring (V033). In the Fatra-Tatra area, where both BINs met, the highest levels of molecular divergence and a relatively clear boundary between the two genetic lineages were found (Fig. 2A). In the studied area, the BIN_W was recorded in a narrower elevation interval compared to BIN_E (Fig. 2B), data from the additional 11 western sites situated above 800 m a.s.l. (Supplementary Table S1) did not reveal presence of *R. tristis* in higher elevation.

**Figure 3:**
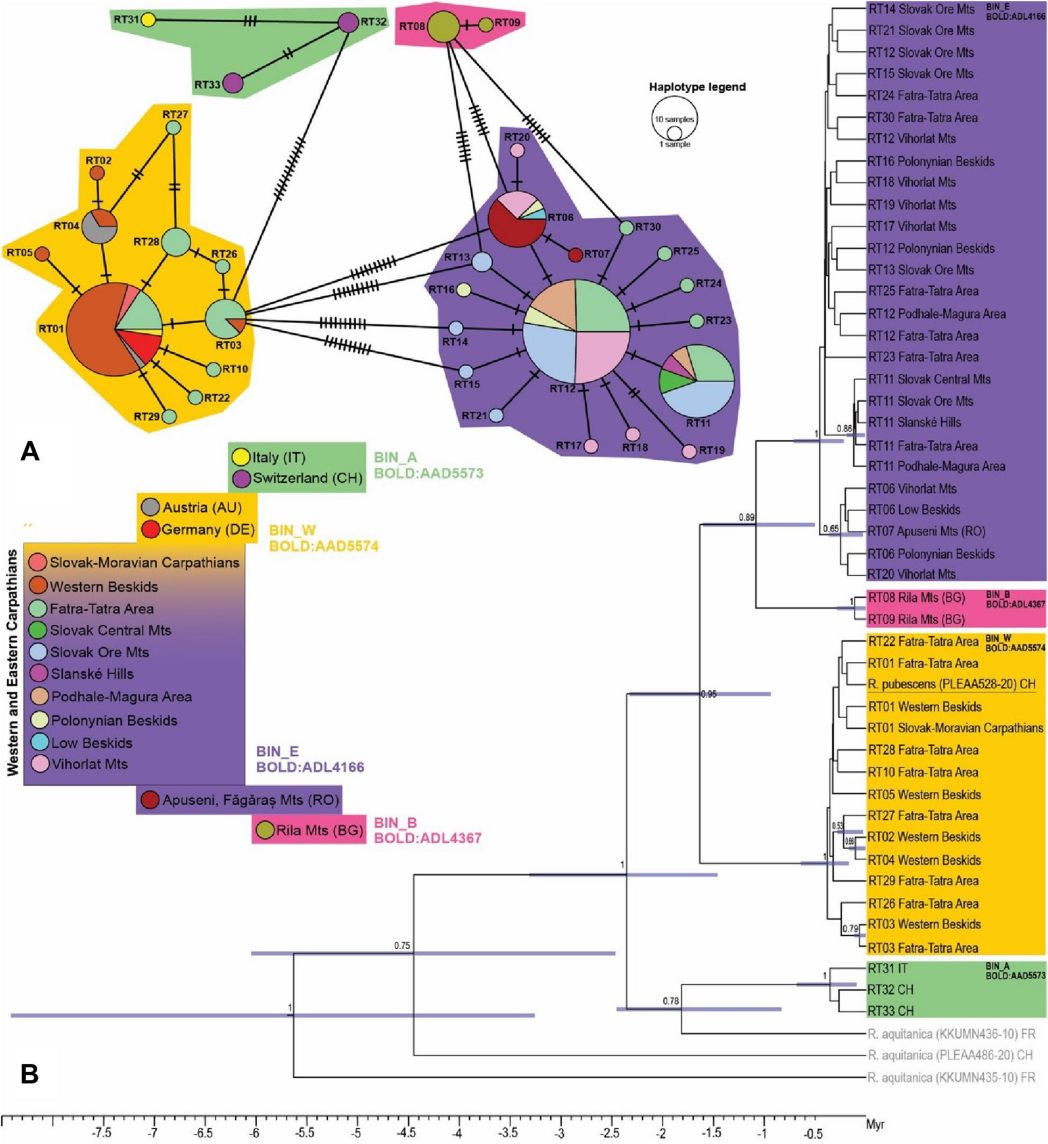
The genetic diversity of the *R. tristis* BINs in the studied area. (**A**) Median-Joining haplotype network (5′ COI mtDNA) showing the relationships among haplotypes RT01–RT33 (including available haplotypes from outside of the studied area). Groups of haplotypes form four BINs (BIN_W, BIN_E, BIN_B, BIN_A), which are colour-coded in the haplotype network according to the legend; (**B**) Bayesian time calibrated phylogenetic reconstruction of *R. tristis* based on COI 5′ region. Numbers near nodes are posterior probabilities. Node bars are 95% HPD of the divergence time. The outgroup consisted of the European congeneric species *R. aquitanica*.

The haplotype network revealed clear relationships between the two BINs identified in the study area as well as their connection with two additional *R. tristis* BINs from other European mountain ranges: the Balkan BIN_B (*BOLD:ADL4367*) occurring, e.g., in the Bulgarian Rila Mts and the BIN_A (*BOLD:AAD5573*) from the Swiss and Italian Alps (Fig. 3A). In total, 28 5′ COI haplotypes of *R. tristis* were identified within 161 individuals from the W Carpathians. The BIN_W included 59 sequences grouped in 11 haplotypes, BIN_E 102 sequences in 17 haplotypes. No significant differences were found in molecular genetic indices between the two BINs (Table 1).

**Table 1:** Molecular diversity of BIN_W and BIN_E lineages of *R. tristis* population in the W Carpathians.

*R. tristis*	H	Π	S	K
BIN_W	0.569	0.00136	9	0.781
BIN_E	0.640	0.00142	16	0.816
Wilcoxon signed rank test BIN_W/BIN_E (p-value)	103 (0.33)	117.5 (0.66)	114.5 (0.58)	117.5 (0.66)

The statistical significance was computed with the Wilcoxon signed rank test for paired data (p-value).

*H* haplotype diversity, *Π* nucleotide diversity, *S* number of polymorphic sites, *K* average number of nucleotide differences.

Both BIN_W and BIN_E showed similar haplotype network patterns, i.e., star-like topologies with a central, the most-frequent haplotype. The most common haplotype in the BIN_W lineage (RT01) was shared with German, Austrian, and Italian localities. This BIN included eight private haplotypes, six of them were found only in the Fatra-Tatra Area and two were private for the Western Beskids. Within the BIN_E, the total number of private haplotypes was 14, four private haplotypes were identified in the Fatra-Tatra Area, the Slovak Ore Mts, and the Vihorlat Mts, one was found in the Poloniny Mts and the Apuseni Mts. The most common haplotype of the BIN_E (RT12 with 55 sequences) occurred at 32 sites. Only a single haplotype (RT06) was shared among the Inner and Outer Eastern Carpathians (PM, VM, LB) and more distant localities in Romania (Fig. 3A). Sequences from Romania were assigned to BIN_E, while sequences from Germany and Austria fell into the western BIN_W. The Bayesian phylogenetic reconstruction of *R. tristis* (5′ COI) revealed that divergence of the species likely started some 2.5 million years ago (Myr) and the western and eastern BINs diverged ca. one and a half million years later. Possible misidentification of sequences (marked as *R. pubescens*) downloaded from public databases was also noted within the analysis (Fig. 3B). Both phylogenetic trees based on 3′ or 5′ COI sequences also showed that the BIN_E is more closely related to the Balkan BIN_B from the remote Rila Mts than to the BIN_W occurring in the same mountain system (Figs. 3B, S1).

The AMOVA of the whole dataset showed that most of the molecular variance could be attributed to the division of samples to physiographic units or to river basins (Table S2). After partitioning the dataset into BIN_W and BIN_E, the highest proportion of the molecular variance was found within sampling localities (= subpopulations) in both BINs (Table 2). In the BIN_W, a relatively large portion of the total variance was explained by the differences among subpopulations within physiographic units (PU) or river basins (RB). In the BIN_W, the variation at this level was negligible, however, influence of the division into PU/RB was suggested (Table 2).

**Table 2:** Analysis of molecular variance (AMOVA) calculated from 161 5′ COI mtDNA sequences of *R. tristis* (BIN_W, BIN_E) from springs and streams in the W Carpathians.

	Source of variation	Df	SS	Variance components	% of variation	F value	p-value
***R. tristis* BIN_W**							
PU	Among PU	2	2.383	0.01838	4.45	F_CT_ = 0.044	0.306
	Among subpopulations within PU	13	10.636	0.17115	41.40	F_SC_ = 0.433	0.001
	Within subpopulations	43	9.625	0.22384	54.15	F_ST_ = 0.459	0.001
RB	Among RB	3	1.472	− 0.05425	− 14.16	F_CT_ = − 0.142	0.888
	Among subpopulations within RB	12	11.548	0.21350	55.73	F_SC_ = 0.488	0.001
	Within subpopulations	43	9.625	0.22384	58.43	F_ST_ = 0.416	0.001
***R. tristis* BIN_E**							
PU	Among PU	7	7.667	0.06698	15.86	F_CT_ = 0.159	0.001
	Among subpopulations within PU	36	12.358	− 0.00986	− 2.33	F_SC_ = − 0.02	0.653
	Within subpopulations	58	21.181	0.36519	86.47	F_ST_ = 0.135	0.087
RB	Among RB	6	7.505	0.06957	16.33	F_CT_ = 0.163	0.001
	Among subpopulations within RB	36	12.443	− 0.00878	− 2.06	F_SC_ = − 0.025	0.574
	Within subpopulations	58	21.181	0.36519	85.73	F_ST_ = 0.143	0.067

The subpopulations are defined as individuals of one sampling site (see Table S3).

*Df* degree of freedom, *SS* sum of squares, *PU* physiographic units, *RB* river basins.

The F_ST_ values indicate a similar range of genetic differentiation within both BINs (Fig. S1). They showed that localities are relatively well connected in both BINs, but some level of isolation could not be refused among several subpopulations. The tests of isolation by distance revealed a positive correlation (Mantel test: r = 0.601; p-value = 0.001), supported also by the significant spatial autocorrelation (p-value = 0.000; Fig. S2). Such a positive and statistically significant correlation suggests a structuring effect of the geographical distance among studied localities.

**Figure 4:**
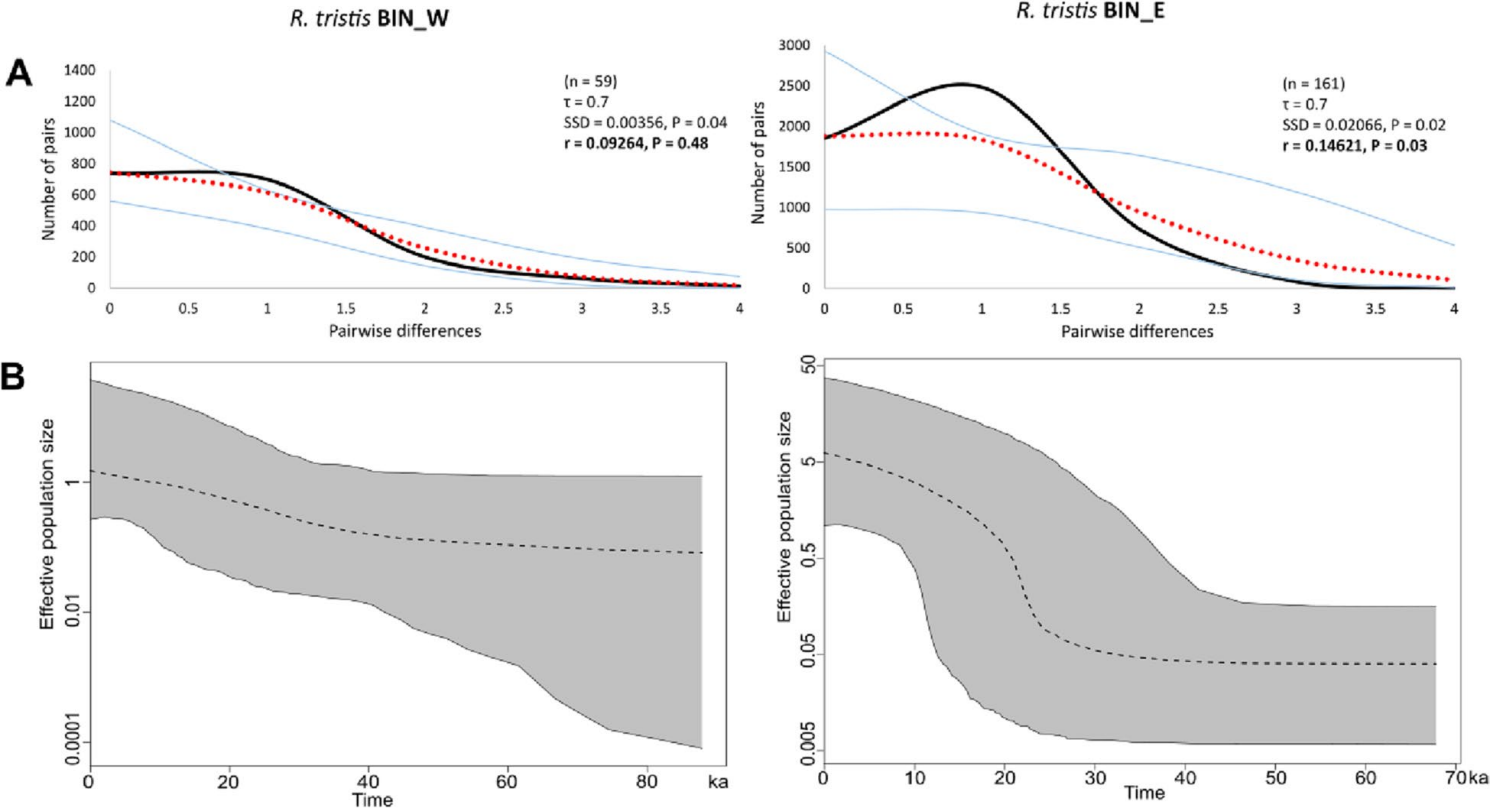
Historical demography of *R. tristis* populations in the W Carpathians. (**A**) Observed unimodal mismatch distribution of BIN_W and BIN_E suggests a recent population expansion. Each plot shows the number (Y axis) of pairwise nucleotide site differences (X axis) among sequences for each BIN. The fit to the demographic expansion model is evaluated by the SSD and the Harpending’s raggedness index (r). The solid black line corresponds to the observed frequency of pairwise differences, the dotted red line represents the pattern expected under a model of sudden demographic expansion. The blue lines are the upper and lower boundaries of the 95% confidence interval; (**B**) Changes in effective population size through time estimated by extended Bayesian Skyline plots, reconstructing the population size history using an evolutionary rate 0.0115 substitutions/site/Myr. The x-axis is depicted on a scale of thousands of years (ka), while Y-axis corresponds to the log mean effective population size. The dotted line represents the mean, while grey-shaded areas encompass 95% central posterior density.

The mismatch distribution analysis suggests a recent population expansion for both BIN_W and BIN_E, a scenario depicted by a typical unimodal distribution (Fig. 4A). In the BIN_W, this was also supported by the non-significant value of the Harpending's raggedness index (r). The values of Tajima's D, Fu's Fs and Fu and Li's D neutrality tests for both BINs were negative and statistically significant (Table 3), which corresponds well with the Mismatch distribution test and supports population expansion hypothesis in both BINs (Fig. 4A). Overall, the results of the neutrality tests of the entire *R. tristis* population had negative values and the values of the Fu's Fs and Fu and Li's D tests were statistically significant (Table 3).

**Table 3:** *R. tristis* mtDNA COI 5′ neutrality tests with p-values for the whole dataset and two different BINs—BIN_W and BIN_E.

Species	Tajima's D test (p-value)	Fu's Fs test (p-value)	Fu and Li's D test (p-value)
*R. tristis*	0.29 (0.71)	− 24.93 (0.000)	− 4.033 (< 0.02)
BIN_W	− 1.62 (0.03)	− 4.788 (< 0.02)	− 5.153 (< 0.02)
BIN_E	− 2.05 (0.00)	− 2.804 (< 0.05)	− 2.751 (< 0.05)

The eBSP also indicates population growth in both *R. tristis* BINs in the study area (Fig. 4B), which likely started between 40–80  ka in BIN_W and ca 25–30 ka in BIN_E. While the population of BIN_W grew gradually, the BIN_E population size increased rapidly in the last 20–25 ka (Fig. 4B).

## Discussion

*Rhyacophila tristis* is a common and widespread upstream species^31^ requiring well-oxygenated water with low organic matter levels^28^. The results of the first European phylogeographic study on this species^29^ showed strong genetic differences between its western and eastern populations, with lineages that probably survived independently in the Pleistocene refugia in the Alps and in the Carpathians. When most of the mountains were covered by ice, populations of some species moved from mountains to adjacent lowland areas and some of them stayed in the isolated areas with suitable environmental conditions. The study of *Asellus aquaticus* (Isopoda) also confirms such survival pattern in the periglacial areas^32^.

Our results show that the presence of the western (BIN_W) and the eastern (BIN_E) lineage was also detected in the W Carpathians themselves, and based on the genetic landscape approach, we proved that W Carpathians form a contact zone between these two lineages (BINs). Reconstruction of phylogeny suggests that the two BINs separated relatively long ago (~ 2.5 Myr, early Pleistocene), indicating (along with p-distance at the level of 2.08%) that in fact they could represent two separate species. However, to make a sound conclusion on their taxonomy, additional studies including more molecular markers as well as morphological feature analysis involving the study of adults would be needed. The analysis also showed that the eastern BIN_E is more closely related to the Balkan samples (BIN_B, Bulgarian Rila Mts) than to the BIN_W co-occuring in the same mountain system (W Carpathians).

Many freshwater species contain endemic genetic lineages or at least private haplotypes occuring in different European mountain systems, including the W Carpathians. For example, the caddisfly *Drusus discolor* very likely persisted in the Tatra Mts in numerous refugia over multiple glacial cycles^23^. Biodiversity richness of the W Carpathians is also supported by studies of the cold-adapted gammarids *Gammarus balcanicus*^4,5^, *G. fossarum*^14^ or *G. jazdzewskii*^33^. Although no endemic lineages of *R. tristis* were found in the W Carpathians, we have detected the presence of 21 private haplotypes within BIN_W and BIN_E, which have not yet been confirmed anywhere else.

The AMOVA of the whole W Carpathian population of *R. tristis* revealed distinct variation among physiographic units and/or river basins. This is, however, very likely due to separate phylogeography, i.e., history and distribution of both detected BINs. On the contrary, the results of AMOVA on the level of individual BINs were more consistent with respect to PU or RB division, which suggests that considering BINs as separate evolutionary units is more natural. In both BINs, the highest portion of the variability was assigned to differences within subpopulations (localities), suggesting relatively recent colonization of the studied area. In the western BIN_W, however, a reasonable part of the genetic variability was attributed to differences among subpopulations within PU/RB, similarly as in aquatic beetle *Elmis aenea* from springs of the same area^34^. On the other hand, AMOVA of BIN_E revealed no variability among localities within PU/RB, but slight differences between PU/RB were detected. This was similar to the more fluvial riffle beetle *Limnius perrisi* from the same study or the blackfly *Simulium degrangei*^35^ studied also in the W Carpathians. F_ST_ values of both BINs agreed with the results of AMOVA. The differences between W Carpathian *R. tristis* lineages on the level of genetic variation between localities within the PU/RB could indicate a lower degree of local isolation and/or higher dispersal ability of BIN_E than BIN_W.

The lineages (BINs) of *R. tristis* from the W Carpathians differed concerning elevation intervals in which they were found. Based on our data, we cannot unambiguously confirm whether this is a stable autecological characteristic of the lineages, because *R. tristis* has a significantly larger area of distribution than the studied area and to confirm or refute the possible difference, we would need more samples. On the other hand, in all sampled localities with an elevation above 800 m a.s.l. in the western part of the studied area, *R. tristis* was missing, which also suggests that the western lineage (BIN_W) could be characteristic for lower altitudes. Elevation influences a series of other factors, such as the proportion of suitable habitats or migration patterns, and indirectly it shapes species population genetics^1^. Moreover, high-elevation sites are often reservoirs of a specific part of the species’ genetic diversity^36–38^, but their vulnerability is high and the loss of isolated populations from higher elevations could result in a loss of important, locally adapted genotypes^39^. Vice versa, adaptation potential and long-term survival of species or genetic lineages are dependent on sufficient intraspecific genetic diversity^40,41^, and from this perspective, i.e., more tolerant to different environmental conditions in different elevations, BIN_E could be at an advantage. On the contrary, if the BIN_W preference for lower elevation is confirmed, this lineage deserves more attention from the point of view of its protection due to higher anthropogenic pressure on lower-situated springs and streams.

By analysis of population genetics, we found the presence of differentiated genetic lineages (BINs) in *R. tristis* and, conversely, similar patterns of distribution of genetic variability as in beetles from the same area^34^, but also in other insect species^36,42^. These findings emphasize the need to study populations in detail at the level of natural evolutionary units. This allows us to better understand the evolution and phylogeography of individual taxa and to look for general trends in the development of the fauna of entire ecosystems.

Beside genetic variability distribution, also the demography of the *R. tristis* lineages detected in the W Carpathians differs. The BIN_W started to expand roughly around 40–80 thousand years ago (ka), while BIN_E around 25 ka during the Last Glacial Maximum (LGM). In that time, latitudinal temperature gradient existed across Europe, when winter soil temperatures were 10–20 °C colder in Central and Northern Europe and around 2–4 °C in Southern Europe than today^43^. Such conditions could suit the up-stream caddisfly *R. tristis*, and so its glacial expansion could be expected^44^. The population of the W Carpathian BIN_E grew sharply compared to BIN_W, and its present effective population size is around five times higher. We assume that this difference could be linked to the different distribution of the BINs. Differences in eBSP and AMOVA could indicate survival of the BIN_W in the study area for a longer period and occurrence of genetic drift after the colonisation of individual sites, causing genetic differences among populations within the PU/RB. In contrast, the results for BIN_E suggest that it spread to the W Carpathians more abruptly and relatively rapidly after LGM. Subtle differences in the variability among PU/RB suggest that individual units/basins were colonized by a part of the originally homogeneous population, which has brought with it specific, although not significantly different compositions of haplotypes. However, the relatively short time of occurrence in the studied area has not yet allowed BIN_E to fix more apparent differences between local populations, as very likely happened within BIN_W.

## Conclusion

The detailed population study of the *R. tristis* caddisfly from the (sub)montane springs and streams in the W Carpathians showed that these habitats form an important and unique type of the freshwater ecosystems producing or harbouring substantial  intraspecific genetic diversity. It can be assumed that these habitats play an equal role also in other areas. The comparison with co-occurring species revealed very interesting results regarding the differences or similarities of their genetic diversity as well as history. This study did not have the ambition to address the taxonomy of the species of interest, but more attention should be paid to this issue in the future, because, as genetic and geographical data have suggested, the possibility that the two identified lineages (BINs) represent separate cryptic species cannot be ruled out. The eastern lineage (BIN_E) most likely colonized the W Carpathians from Southeast Europe, probably at the end of the LGM, while BIN_W appears to originate in areas west of the W Carpathians and it survived and developed its population in the study area for a longer time. The Western Carpathians are the place where the two lineages meet. What role do W Carpathian springs and streams play in survival and preservation of *R. tristis* genetic diversity, whether the lineages hybridize in the contact zone or what are their specific ecological requirements, will need further study including samples from a wider area of distribution and additional genetic markers.

## Materials and methods

### Study area

The study area includes the W Carpathians, one of the major physiographic units of the Carpathian mountain range (Fig. 1A). It covers approximately one-third of the total area of the Carpathians^45^. Most of the W Carpathian ranges are of moderate elevation, ranging mostly from 500 to 1300 m a.s.l., only sparsely exceeding 1500 m a. s. l.^45^. Geologically, the W Carpathians are diversified, especially in the inner part of the arc, with various kinds of bedrock (Mesozoic limestones and dolomites, Paleozoic granites and metamorphic rocks or Tertiary volcanic rocks). The outer W Carpathians are built almost solely from flysch^46^. During the Pleistocene cycles, this area remained mostly unglaciated. The continental ice sheet was only located in the northernmost foothills of the Polish part during its maximal extent in the Middle Pleistocene and the mountain glaciers covered valleys in the highest ranges (above 1700 m a.s.l.)^47^. The mountain glaciers completely disappeared around 8.500 years ago^48^. The precipitation in the area is determined by differences in elevation and geomorphological relief. Hydrologically, the W Carpathian rivers have a rain-snow regime with floods in springs and summers.

The studied localities include 15 springs and 43 streams situated mainly in Slovakia, partially in Czechia and Poland, within the Inner and Outer Western Carpathian physiographic units/subunits. These localities were supplemented by sampling sites in the Inner Eastern Carpathians (Vihorlat Mts—VM, Poloniny Mts—PM) and the Outer Eastern Carpathians (Low Beskids—LB) (Fig. 1B; Table S3).

### Sampling and morphological identification

The qualitative sampling of macrozoobenthos was performed in 2016–2017, within the framework of a broader hydrobiological research focused on karst springs and diversity of the W Carpathian streams. It was carried out by a multi-habitat kick-sampling technique^49^, using a hydrobiological hand-net with a mesh size of 0.5 mm. In the field, specimens were fixed in 96% ethanol. In the laboratory, the invertebrates were sorted into higher taxonomic groups using stereomicroscope, prefixed with clean ethanol and stored in a freezer at − 25 °C. Samples were supplemented by additional specimens of *R. tristis* from the ZooLab collection of the Plant Science and Biodiversity Centre, Slovak Academy of Sciences (Bratislava). The individuals of *R. tristis* were morphologically identified using the determination keys Sedlák from 1980^50^ and Waringer and Graf from 2011^51^.

### DNA extraction and PCR amplification

DNA was extracted from a total of 161 individuals, two legs of each individual, using the Chelex protocol^52^, followed by PCR amplification of ca. 650 bp-long barcoding fragment of the mitochondrial cytochrome c oxidase subunit I (5′ COI) using the primer pair LCO1490–HCO2198^53^. The PCR was performed in a total volume of 25 μl containing 5 μl of 5 × DreamTaq™ Buffer, 1.5 μl of Mg^2+^ (25 mM), 0.5 μl of each primer (concentration 5 mM), 0.5 μl of dNTP Mix (20 mM), 0.125 μl (0.625 U) DreamTaq DNA Polymerase, 11.875 μl ultra-pure H_2_O and 5 μl of DNA template. The PCR cycling consisted of a 2-min initial denaturation at 94 °C, followed by 40 cycles of 94 °C (40 s) denaturation, 46 °C (40 s) annealing and 72 °C (1 min) extension and termination at 72 °C (10 min) for a final extension. A 4 μl aliquot of the PCR products were visualized by GoldView (Solarbio) in electrophoresis on a 1% agarose gel and GelLogic imaging equipment to check PCR products quality and length. The PCR products were purified with Exo-FastAP Thermo Scientific and were sent for sequencing to Macrogen Europe Inc., Amsterdam.

Additionally, for the purpose of linking our data with previous studies of Bálint et al. from 2011^29^ and 2009^54^, the non-barcoding 3′ COI marker using primers Jerry and Pat^55^ was amplified. We sequenced the necessary number of individuals (six specimens selected from different physiographic units) to create a phylogenetic tree together with 19 sequences (3′ COI) of *R. tristis* and 15 sequences (3′ COI) of congeneric species *R. aquitanica* McLachlan, 1879 (8) and *R. carpathica* Botoșaneanu, 1995 (7) from Bálint et al. 2011^29^ and 2009^54^ (Fig. S3).

### Data analysis

The obtained sequences were edited in SEQUENCHER v5.1 and aligned using the MUSCLE algorithm^56^ in MEGA v7^57^. In total, the 5′ COI dataset consisted of 194 sequences of *R. tristis*. It included 161 sequences from the W Carpathians (46 localities), the Poloniny Mts (3 loc.), the Vihorlat Mts (7 loc.) and Low Beskids (1 loc.) (Fig. 1). They were supplemented by 33 reference sequences of *R. tristis* from outside the W Carpathians (5—Austria, 6—Bulgaria, 5—Germany, 2—Italy, 11—Romania, 4—Switzerland), used for the reconstruction of haplotype networks and phylogenetic tree. These sequences were obtained from the BOLD (http://www.boldsystems.org).

Two methods were used to test *R. tristis* samples for presence of the deeper (genetic/evolutionary) lineages. The first was the BIN algorithm implemented in BOLD (http://www.boldsystems.org), where every uploaded sequence is compared to all records and assigned to an existing or a newly created Barcode Index Number (BIN)^58^. The BIN system clusters are unique and include sequences that presumably represent a single species. The second approach utilized the Assemble Species by Automatic Partitioning (ASAP), a novel and powerful method to build species partitions, based on pairwise genetic distances from single locus sequence alignments (i.e., barcode data sets)^59^.

The haplotype data files and the diversity indices were generated in DnaSP v5.10^60^ based on 5′ COI data. We calculated haplotype diversity (H), nucleotide diversity (π), number of polymorphic sites (S) and average number of nucleotide differences (K) per individual BINs of *R. tristis* species within the W Carpathians. Statistical comparison of the genetic indices between BINs was computed with the Wilcoxon signed rank test for paired data in R v4.0.2 (http://www.r-project.org).

Haplotype networks were reconstructed using the median-joining method (MJN) in PopART v1.7^61^. The networks include sequences outside the W Carpathians to explain the phylogeographic relationships and haplotype distribution in the broader context.

The phylogeny was reconstructed based on 5′ COI haplotypes using Bayesian approach in BEAST v2.5^62^ and, separately, for the 3′ COI marker also including published sequences^29,54^. As an outgroup, the European congeners *R. aquitanica* and *R. carpathica* were included. The nucleotide substitution model was set through bModelTest^63^. The tree prior was set to Birth–Death and a strict molecular clock was used following the Path Sampling Selection. The strict molecular clock was calibrated with the standard mitochondrial rate for arthropod COI equal to 0.0115 substitutions/site/Myr^64^. Two runs of Markov chain Monte Carlo (MCMC), each 20 million iterations long and sampled every 1000 iterations, were performed for both fragments. Runs were examined using Tracer v1.7^65^, and all sampled parameters achieved a sufficient sample size (ESS > 200). Tree files were combined using Log-Combiner v2.5.2^62^, with the removal of the non-stationary 25% burn-in phase. The maximum clade credibility chronogram was generated using TreeAnnotator v2.5.2^62^. The same methodology was used in case of the non-barcoding 3′ COI marker phylogeny reconstruction (Fig. 3).

The spatial diversity pattern in the studied area of Carpathians was illustrated with a genetic landscape approach generated with Alleles in Space (AIS)^66^. The genetic landscape visualizes the abrupt transitions between populations and groups of populations characterized by divergent haplotypes. First, using the AIS software, genetic distances between localities were calculated based on all 5′ COI sequences and connected into a network based on the Delaunay Triangulation. The genetic distance values were set in the midpoints of each connection in the network. The raw genetic distances acquired were interpolated afterwards and the matrix of the ‘elevation’ values, with their respective latitude and longitude coordinates, was then imported into QGIS 3.16 (https://qgis.org/) software to produce a genetic divergence surface image using the inverse distance weighted algorithm. The resulting image was plotted onto a relief map to create a final map in which the hypsometric tints (red-blue) reflect the genetic distance between population pairs.

To test if spatial distance is structuring the molecular diversity of 5′ COI fragments, we run two types of isolation by distance tests: Mantel test^67^ and general spatial autocorrelation test using Alleles in Space (AIS)^66^. Both tests analyse correlation between spatial and molecular distance. To assess the significance, tests were run with 1000 permutations.

The population structure of *R. tristis* in the W Carpathians based on 5′ COI was characterized by the analysis of molecular variance (AMOVA) and fixation indices (F_ST_) using Arlequin v3.5^68^. The AMOVA was used to estimate whether the observed genetic diversity may be attributed to the geographical or river basin partitioning of populations in three levels: among physiographic units (PU)/river basins (RB), among sampling sites within PU/RB and within sampling sites. We also performed AMOVA and F_ST_ separately for individual BINs of the *R. tristis*, to find out the differences between them. 138 sequences of *R. tristis* (36 localities) were included to calculate the F_ST_ index, localities with only 1 sequence were excluded. To test the significance of covariance components and fixation indices, 1,000 permutations were performed.

Historical expansion patterns of the *R. tristis* in the studied area of Carpathians, based on 5′ COI, were examined in three different approaches. First, Tajima’s D^69^, Fu’s Fs^70^ and Fu and Li’s D^71^ neutrality tests with 10,000 permutations were calculated in DnaSP v5.10 to test the selective neutrality and population stability. Secondly, nucleotide mismatch distribution was performed in Arlequin v3.5^68^ to detect the demographic and spatial dynamics of population expansion history in the W Carpathians. The fit between the observed and expected distributions was evaluated by the test statistics of goodness-of-fit, including the sum of squared deviation (SSD) and Harpending’s raggedness index (r).

Finally, the extended Bayesian skyline plot (eBSP), implemented in BEAST v2.6.2 software package ^62^, was used to show the fluctuations of *R. tristis* demography in the W Carpathians over time. The model of substitution and molecular clock were set up identical as in the case of the phylogeny reconstruction. For comparison, two runs of Monte Carlo Markov Chains (MCMC) were performed, each 40 million iterations long and sampled every 10,000 iterations. The runs were examined in Tracer v1.7 and all the parameters reached the effective sampling size (ESS) above 200. The eBSP plots were produced using R v4.0.2 software (http://www.r-project.org). Both runs showed complementary results, thus only one is presented.

## Supplementary Information

Supplementary Information.

## Data Availability

All sequences used in this study are publicly available on GenBank (accession numbers: FJ514783-FJ514797, GU713149, GU713174, HM204653, HM204660, HM204668, HM204670-HM204672, HM204675, HM204678, HM204684, HM204686, HM204689-HM204691, HM395967, HM401417, HM862489, KX102651, KX142737, KX143092, KX291844, KX294817, KY262567, OK623503-OK623511, OK623926-OK624110) and are included in the BOLD dataset DS-SKRHYTRI under the 10.5883/DS-SKRHYTRI.

## References

[1] Manel S, Schwartz MK, Luikart G, Taberlet P (2003). Landscape genetics: Combining landscape ecology and population genetics. Trends Ecol. Evol..

[2] Storfer A, Murphy MA, Spear SF, Holderegger R, Waits LP (2010). Landscape genetics: Where are we now?. Mol. Ecol..

[3] Alp M, Keller I, Westram AM, Robinson CT (2012). How river structure and biological traits influence gene flow: A population genetic study of two stream invertebrates with differing dispersal abilities. Freshw. Biol..

[4] Mamos T, Wattier R, Majda A, Sket B, Grabowski M (2014). Morphological vs. molecular delineation of taxa across montane regions in Europe: The case study of *Gammarus balcanicus* Schäferna, 1922 (Crustacea: Amphipoda). J. Zool. Syst. Evol. Res..

[5] Mamos T, Wattier R, Burzýnski A, Grabowski M (2016). The legacy of a vanished sea: A high level of diversification within a European freshwater amphipod species complex driven by 15 My of Paratethys regression. Mol. Ecol..

[6] Grabowski M, Mamos T, Bacela-Spychalska K, Rewicz T, Wattier RA (2017). Neogene paleogeography provides context for understanding the origin and spatial distribution of cryptic diversity in a widespread balkan freshwater amphipod. PeerJ.

[7] Copilaş-Ciocianu D, Zimţa AA, Grabowski M, Petrusek A (2018). Survival in northern microrefugia in an endemic Carpathian gammarid (Crustacea: Amphipoda). Zool. Scr..

[8] Copilaș-Ciocianu D, Zimța A, Petrusek A (2019). Integrative taxonomy reveals a new *Gammarus* species (Crustacea, Amphipoda) surviving in a previously unknown southeast European glacial refugium. J. Zool. Syst. Evol. Res..

[9] Wattier R, Mamos T, Copilaş-Ciocianu D, Jelić M, Ollivier A, Chaumot A, Danger M, Felten V, Piscart C, Žganec K, Rewicz T, Wysocka A, Rigaud T, Grabowski M (2020). Continental-scale patterns of hyper-cryptic diversity within the freshwater model taxon *Gammarus fossarum* (Crustacea, Amphipoda). Sci. Rep..

[10] Neumann K, Michaux JR, Maak S, Jansman HA, Kayser A, Mundt G, Gattermann R (2005). Genetic spatial structure of European common hamsters (*Cricetus cricetus*)—A result of repeated range expansion and demographic bottlenecks. Mol. Ecol..

[11] Kotlík P, Deffontaine V, Mascheretti S, Zima J, Michaux JR, Searle JB (2006). A northern glacial refugium for bank voles (*Clethrionomys glareolus*). PNAS.

[12] Theissinger K, Bálint M, Feldheim KA, Haase P, Johannesen J, Laube I, Pauls SU (2012). Glacial survival and post-glacial recolonization of an arctic-alpine freshwater insect (*Arcynopteryx dichroa*, Plecoptera, Perlodidae) in Europe. J. Biogeogr..

[13] Vörös J, Mikulíček P, Major Á, Recuero E, Arntzen JW (2016). Phylogeographic analysis reveals northern refugia for the riverine amphibian *Triturus dobrogicus* (Caudata: Salamandridae). Biol. J. Linn. Soc..

[14] Copilaș-Ciocianu D, Rutová T, Pařil P, Petrusek A (2017). Epigean gammarids survived millions of years of severe climatic fluctuations in high latitude refugia throughout the Western Carpathians. Mol. Phylogenet. Evol..

[15] Juřičková L, Pokorný P, Hošek J, Horáčková J, Květoň J, Zahajská P, Jansová A, Ložek V (2017). Early postglacial recolonisation, refugial dynamics the origin of a major biodiversity hotspot. A case study from the Malá Fatra mountains, Western Carpathians, Slovakia. Holocene.

[16] Mamos T, Jażdżewski K, Čiamporová-Zaťovičová Z, Čiampor F, Grabowski M (2021). Fuzzy species borders of glacial survivalists in the Carpathian biodiversity hotspot revealed using a multimarker approach. Sci. Rep..

[17] Pinceel J, Jordaens K, Pfenninger M, Backeljau T (2005). Rangewide phylogeography of a terrestrial slug in Europe: Evidence for Alpine refugia rapid colonization after the Pleistocene glaciations. Mol. Ecol..

[18] Magri D, Fineschi S, Bellarosa R, Buonamici A, Sebastiani F, Schirone B, Simeone MC, Vendramin GG (2006). A new scenario for the Quaternary history of European beech populations: Palaeobotanical evidence genetic consequences. New Phytol..

[19] Jamrichová E, Potůčková A, Horsák M (2014). Landscape history, calcareous fen development historical events in the Slovak Eastern Carpathians. Veg. Hist. Archaeobot..

[20] Jamrichová E, Petr L, Jiménez-Alfaro B (2017). Pollen-inferred millennial changes in landscape patterns at a major biogeographical interface within Europe. J. Biogeogr..

[21] Wielstra B, Babik W, Arntzen JW (2015). The crested newt *Triturus cristatus* recolonized temperate Eurasia from an extra-Mediterranean glacial refugium. Biol. J. Linn. Soc..

[22] Mráz P, Ronikier M (2016). Biogeography of the Carpathians: Evolutionary spatial facets of biodiversity. Biol. J. Linn. Soc..

[23] Pauls SU, Lumbsch HAT, Haase P (2006). Phylogeography of the montane caddisfly *Drusus discolor*: Evidence for multiple refugia and periglacial survival. Mol. Ecol..

[24] Pauls SU, Theissinger K, Ujvarosi L, Bálint M, Haase P (2009). Patterns of population structure in two closely related, partially sympatric caddisflies in eastern Europe: Historic introgression, limited dispersal, and cryptic diversity. J. N. Am. Benthol. Soc..

[25] Lehrian S, Pauls SU, Haase P (2009). Contrasting patterns of population structure in the montane caddisflies *Hydropsyche tenuis* and *Drusus discolor* in the Central European highlands. Freshw. Biol..

[26] Lande R, Shannon S (1996). The role of genetic variation in adaptation and population persistence in a changing environment. Evolution.

[27] Frankham R, Briscoe DA, Ballou JD (2002). Introduction to Conservation Genetics.

[28] Robert S, Curtean-Bănăduc A (2005). Aspects concerning Târnava Mare and Târnava Mică rivers (Transylvania, Romania) caddisfly (Insecta, Trichoptera) larvae communities. Transylv. Rev. Syst. Ecol. Res..

[29] Bálint M, Ujvárosi L, Dénes AL, Octavian P (2011). European phylogeography of *Rhyacophila tristis* Pictet (Trichoptera: Rhyacophilidae): Preliminary results. Zoosymposia.

[30] Bielik M (1999). Geophysical features of the Slovak Western Carpathians. Geol. Q..

[31] Céréghino R, Cugny P, Lavandier P (2002). Influence of intermittent hydropeaking on the longitudinal zonation patterns of benthic invertebrates in a mountain stream. Int. Rev. Hydrobiol..

[32] Sworobowicz L, Mamos T, Grabowski M, Wysocka A (2020). Lasting through the ice age: The role of the proglacial refugia in the maintenance of genetic diversity, population growth, and high dispersal rate in a widespread freshwater crustacean. Freshw. Biol..

[33] Rudolph K, Coleman CO, Mamos T, Grabowski M (2018). Description and post-glacial demography of *Gammarus jazdzewskii* sp. nov. (Crustacea: Amphipoda) from Central Europe. Syst. Biodivers..

[34] Bozáňová J, Čiamporová-Zaťovičová Z, Čiampor F, Mamos T, Grabowski M (2020). The tale of springs and streams: How different aquatic ecosystems impacted the mtDNA population structure of two riffle beetles in the Western Carpathians. PeerJ.

[35] Jedlička L, Kúdela M, Szemes T, Celec P (2012). Population genetic structure of *Simulium degrangei* (Diptera: Simuliidae) from Western Carpathians. Biologia.

[36] Hughes JM, Bunn SE, Hurwood DA, Cleary C (1998). Dispersal and recruitment of *Tasiagma ciliata* (Trichoptera: Tasmiidae) in rainforest streams, south-east Queensland, Australia. Freshw. Biol..

[37] Finn DS, Theobald DM, Black WC, Poff NL (2006). Spatial population genetic structure and limited dispersal in a Rocky Mountain alpine stream insect. Mol. Ecol..

[38] Vuataz L, Rutschmann S, Monaghan MT, Sartori M (2016). Molecular phylogeny and timing of diversification in Alpine *Rhithrogena* (Ephemeroptera: Heptageniidae). BMC Evol. Biol..

[39] Schiffers K, Bourne EC, Lavergne S, Thuiller W, Travis JMJ (2013). Limited evolutionary rescue of locally adapted populations facing climate change. Philos. Trans. R. Soc. B Biol. Sci..

[40] Spielman D, Brook B, Frankham R (2004). Most species are not driven to extinction before genetic factors impact them. Proc. Natl. Acad. Sci..

[41] Frankham R (2005). Genetics and extinction. Biol. Conserv..

[42] Bunn SE, Hughes JM (1997). Dispersal and recruitment in streams: Evidence from genetic studies. J. N. Am. Benthol. Soc..

[43] Barron E, Pollard D (2002). High-resolution climate simulations of oxygen isotope stage 3 in Europe. Quat. Res..

[44] Bennet K, Provan J (2008). What do we mean by “refugia”?. Quat. Sci. Rev..

[45] Kondracki, J. Karpaty. Wydanie drugie i poprawione [The Carpathians. Ed. 2].—Wydawnictwa Szkolne i Pedagogiczne, Warszawa (1989).

[46] Grecula, P. (ed.). Geological evolution of the Western Carpathians. Monograph: Mineralia Slovaca (1997).

[47] Lukniš M (1964). The course of the last glaciation of the Western Carpathians in the relation to the Alps, to the glaciation of northern Europe, and to the division of the central European Wurm into periods. Geografický Časopis.

[48] Lindner L, Dzierzek J, Marciniak B, Nitychoruk J (2003). Outline of Quaternary glaciations in the Tatra Mts.: Their development, age and limits. Geol. Q..

[49] Frost S (1971). Evaluation of kicking technique for sampling stream bottom fauna. Can. J. Zool..

[50] Sedlák E, Rozkošný R (1980). Řád Chrostíci—Trichoptera. Klíč vodních larev hmyzu.

[51] Waringer J, Graf W (2011). Atlas of Central European Trichoptera Larvae: Atlas der Mitteleuropäischen Köcherfliegenlarven.

[52] Casquet J, Thebaud C, Gillespie RG (2012). Chelex without boiling, a rapid and easy technique to obtain stable amplifiable DNA from small amounts of ethanol-stored spiders. Mol. Ecol. Resour..

[53] Folmer O, Black M, Hoeh W, Lutz R, Vrijenhoek R (1994). DNA primers for amplification of mitochondrial cytochrome c oxidase subunit I from diverse metazoan invertebrates. Mol. Mar. Biol. Biotechnol..

[54] Bálint M, Botoşaneanu L, Ujvárosi L, Popescu O (2009). Taxonomic revision of *Rhyacophila aquitanica* (Trichoptera: Rhyacophilidae), based on molecular and morphological evidence and change of taxon status of *Rhyacophila aquitanica* ssp. carpathica to *Rhyacophila carpathica* stat. n. Zootaxa.

[55] Simon C, Frati F, Beckenbach A, Crespi B, Liu H, Flook P (1994). Evolution, weighting and phylogenetic utility of mitochondrial gene sequences and a compilation of conserved polymerase chain reaction primers. Ann. Entomol. Soc. Am..

[56] Edgar RC (2004). MUSCLE: Multiple sequence alignment with high accuracy and high throughput. Nucleic Acids Res..

[57] Kumar S, Stecher G, Tamura K (2016). MEGA7: Molecular evolutionary genetics analysis version 7.0 for bigger datasets. Mol. Biol..

[58] Ratnasingham S, Hebert PDN (2007). The barcode of life data system. Mol. Ecol. Notes.

[59] Puillandre N, Brouillet S, Achaz G (2021). ASAP: Assemble species by automatic partitioning. Mol. Ecol. Resour..

[60] Librado P, Rozas J (2009). DnaSP v5: A software for comprehensive analysis of DNA polymorphism data. Bioinformatics.

[61] Leigh JW, Bryant D (2015). POPART: Full-feature software for haplotype network construction. Methods Ecol. Evol..

[62] Bouckaert R, Vaughan TG, Barido-Sottani J, Duchêne S, Fourment M, Gavryushkina A, Heled J, Jones G, Kühnert D, De Maio N, Matschiner M, Mendes FK, Müller NF, Ogilvie HA, duPlessis L, Popinga A, Rambaut A, Rasmussen D, Siveroni I, Suchard MA, Wu C-H, Xie D, Zhang C, Stadler T, Drummond AJ (2019). BEAST 2.5: An advanced software platform for Bayesian evolutionary analysis. PLoS Comput. Biol..

[63] Bouckaert RR, Drummond AJ (2017). bModelTest: Bayesian phylogenetic site model averaging and model comparison. BMC Evol. Biol..

[64] Brower AVZ (1994). Rapid morphological radiation and convergence among races of the butterfly *Heliconius erato* inferred from patterns of mitochondrial DNA evolution. PNAS.

[65] Rambaut A, Drummond AJ, Xie D, Baele G, Suchard MA (2018). Posterior summarisation in Bayesian phylogenetics using Tracer 1.7. Syst. Biol..

[66] Miller MP (2005). Alleles In Space (AIS): Computer software for the joint analysis of interindividual spatial and genetic information. J. Hered..

[67] Mantel N (1967). The detection of disease clustering and a generalized regression approach. Cancer Res..

[68] Excoffier L, Lischer HE (2010). Arlequin suite ver 3.5: A new series of programs to perform population genetics analyses under Linux and Windows. Mol. Ecol. Resour..

[69] Tajima F (1989). The effect of change in population size on DNA polymorphism. Genetics.

[70] Fu YX (1997). Statistical tests of neutrality of mutations against population growth, hitchhiking and background selection. Genetics.

[71] Fu YX, Li WH (1993). Statistical tests of neutrality of mutations. Genetics.

