# Supplementary material for: DNA barcodes evidence the contact zone of eastern and western caddisfly lineages in the Western Carpathians
Source: Sci Rep. 2021 Dec 15;11:24020. doi: 10.1038/s41598-021-03411-8 (PMC8674257; doi:10.1038/s41598-021-03411-8)
Supplement: Supplementary file 1 — Supplementary Information. [file 41598_2021_3411_MOESM1_ESM.docx]

**Supplementary Materials**

**DNA barcodes evidence the contact zone of eastern and western caddisfly lineages in the Western Carpathians**

**Jana Bozáňová^1,2^, Fedor Čiampor Jr^1^, Tomasz Mamos^3^, Michal Grabowski^3^, Zuzana Čiamporová-Zat'ovičová^1,2,*^**

^1^ ZooLab, Plant Science and Biodiversity Centre, Slovak Academy of Sciences, Dúbravská cesta 9, 845 23 Bratislava, Slovak Republic

^2^ Department of Ecology, Faculty of Natural Sciences, Comenius University in Bratislava, Ilkovičova 6, 842 15 Bratislava, Slovak Republic

^3^ Department of Invertebrate Zoology and Hydrobiology, Faculty of Biology & Environmental Protection, University of Lodz, Banacha 12/16,90-237 Lodz, Poland

* Corresponding author: [zuzana.zatovicova@savba.sk](mailto:zuzana.zatovicova@savba.sk)

**
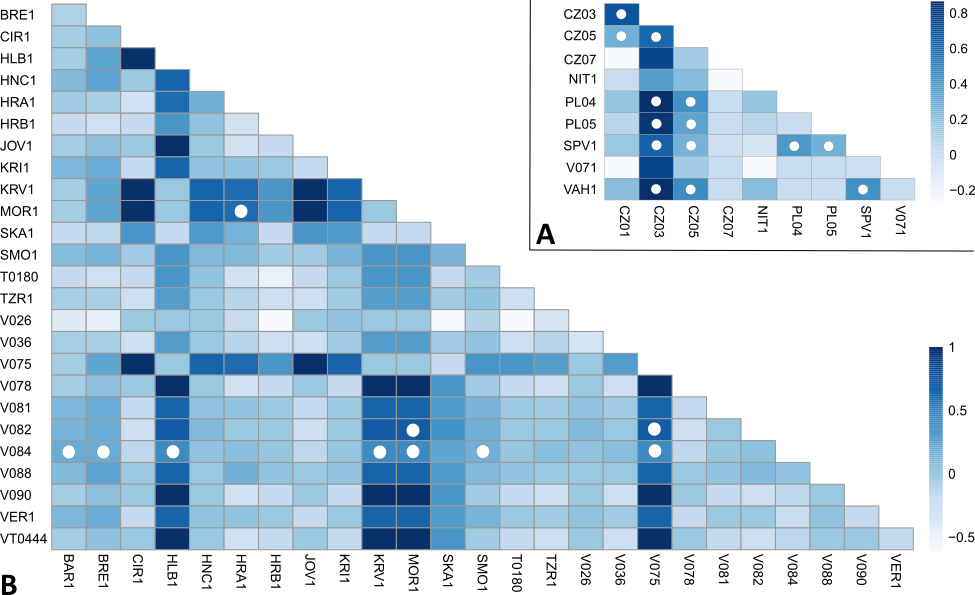
Supplementary Figure S1.** Heat map of pairwise F_ST_ values among studied sites (subpopulations) of *R. tristis* in the W Carpathians, (A) BIN_W, (B) BIN_E. Darker blue shades indicate higher F_ST_ values (as displayed on the bar right of the map). White dots indicate F_ST_ p-values significantly different from zero (p-value < 0.05).


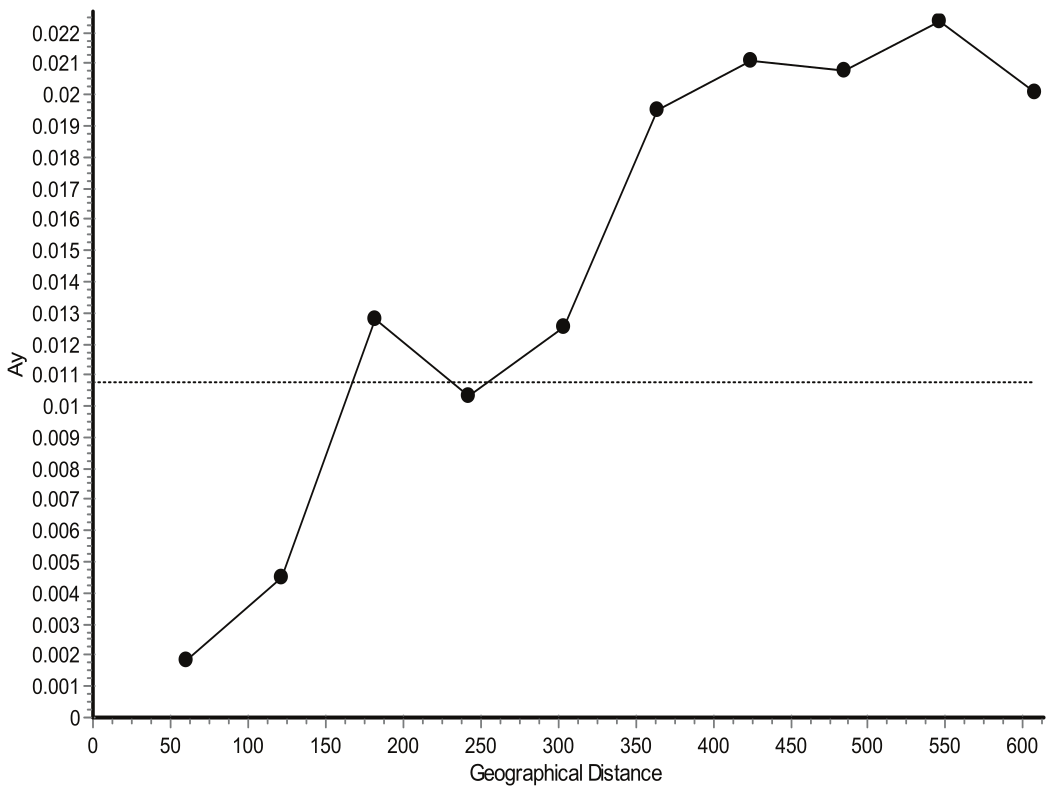


**Supplementary Figure S2.** Results of the spatial autocorrelation analysis of the *R. tristis* (P = 0.000). Distance classes (10) are given on the x-axis between 0 and 613 km, and pairwise genetic distances (Ay) are given on the y-axis. The horizontal dotted line indicates the average genetic distance for the observed data set. Values above the horizontal line suggest a correlation between distance class and genetic distance.

**
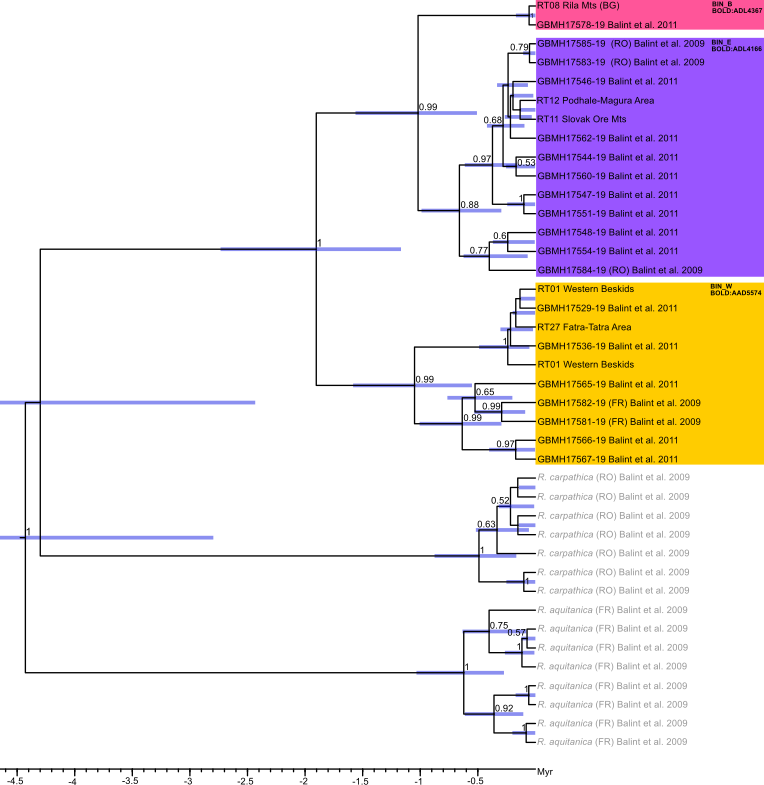
Supplementary Figure S3.** Bayesian time calibrated phylogenetic reconstruction of the W Carpathian sequences and *R. tristis* sequences from Bálint et al. in 2011^[29]^ (3' COI mtDNA) together with the outgroup species *R. aquitanica* (FR) and *R. carpathica* (RO). Abbreviations: Bulgaria (BG), Romania (RO), France (FR).

| **Mts System** | **Geomorphological Unit** | **Geomorphological Subunit** | **Code** | **Locality** | **Habitat** | **River basin** | **GPS Coordinates** | **Altitude** |
| --- | --- | --- | --- | --- | --- | --- | --- | --- |
| **IWC** | **FTA**  Fatra-Tatra Area  (Fatránsko-tatranská oblasť) | **FTA1**  Tatras (Tatry) | **V023** | Pri salaši | SP | Váh | 49.0845, 19.9457 | 840 m |
|  |  |  | **V024** | Pod Hrubým Grúňom | SP | Váh | 49.0863, 19.9441 | 820 m |
|  |  |  | **V025** | Brestovská | SP | Váh | 49.2586,19.6576 | 851 m |
|  |  |  | **V069** | Bobrovecká | SP | Váh | 49.2643,19.7589 | 1961 m |
|  |  | **FTA2**  Belianske Tatras (Belianske Tatry) | **V035** | Pod Štefanom | SP | Váh | 49.2422, 20.1626 | 1116 m |
|  |  |  | **V036** | Dolina 7 prameňov | SP | Váh | 49.2228, 20.2776 | 1208 m |
|  |  | **FTA3**  Great Fatra (Veľká Fatra) | **V030** | Jazierce | SP | Váh | 49.0182, 19.2819 | 589 m |
|  |  | **FTA4**  Little Fatra (Malá Fatra) | **V094** | Mojžišov 1 | SP | Váh | 48.6624, 19.6285 | 1147 m |
|  |  |  | **VO95** | Mojžišov 2 | SP | Váh | 49.1905, 19.0726 | 1147 m |
| **OWC** | **CB**  Central Beskids  (Stredné Beskydy) | **CB1**  Kysucké Beskydy | **OSC1** | Oščadnica | ST | Váh | 49.4212, 18.9108 | 822 m |
|  | **WB**  Western Beskids  (Západné Beskydy) | **WB1**  Moravian-Silesian Beskids  (Moravsko-sliezske Beskydy) | **CZ04** | Černa Ostravice | ST | Morava | 49.4566, 18.4709 | 816 m |

**Supplementary Table S1.** Additional 11 western sites situated above 800 m a.s.l. did not reveal the presence of BIN_W of *R. tristis* in higher elevation. IWC – Inner Western Carpathians, OWC – Outer Western Carpathians; SP – spring, ST – stream.

|  | ***R. tristis*** |  |  |  |  |  |  |
| --- | --- | --- | --- | --- | --- | --- | --- |
|  | **Source of variation** | **Df** | **SS** | **Variance components** | **% of variation** | **F value** | **p-value** |
| **PU** | Among PU | 9 | 288.568 | 1.99019 | 56.53 | F_CT_ = 0.565 | > 0.00 |
|  | Among subpopulations within PU | 48 | 156.986 | 1.00795 | 28.63 | F_SC_ = 0.659 | > 0.00 |
|  | Within subpopulations | 104 | 54.354 | 0.52263 | 14.84 | F_ST_ = 0.852 | < 0.00 |
| **RB** | Among RB | 8 | 351.688 | 2.46668 | 70.03 | F_CT_ = 0.700 | > 0.00 |
|  | Among subpopulations within RB | 48 | 92.219 | 0.53299 | 15.13 | F_SC_ = 0.505 | > 0.00 |
|  | Within subpopulations | 104 | 54.354 | 0.52263 | 14.84 | F_ST_ = 0.852 | < 0.00 |

**Supplementary Table S2.** Analysis of molecular variance (AMOVA) calculated from 161 COI mtDNA sequences of *R. tristis* from studied springs and streams in the W Carpathians. The subpopulation is defined as individuals of one sampling site (see Table S3). Df – degree of freedom, SS – sum of squares, PU – physiographic units, RB – river basins.

| **Mts System** | **Physiographic Unit** | **Physiographic Subunit** | **Code** | **Locality** | **Habitat** | **River basin** | **GPS Coordinates** | **Altitude** | **BIN** |
| --- | --- | --- | --- | --- | --- | --- | --- | --- | --- |
| **IWC** | **FTA**  Fatra-Tatra Area  (Fatransko-tatranská oblasť) | **FTA1**  Little Carpathians (Malé Karpaty) | V005 | Doľany | SP | Morava | 48.4245, 17.3210 | 424 m | BIN_W(1) |
|  |  |  | V071 | Husí stok | SP | Morava | 48.4651, 17.3767 | 273 m | BIN_W(8) |
|  |  |  | SPV1 | Stupavský p. 1 | ST | Morava | 48.2525, 17.1122 | 292 m | BIN_W(8) |
|  |  | **FTA2**  Tatras (Tatry) | T180 | Biela voda | ST | Dunajec a Poprad | 49.2291, 20.0999 | 1065 m | BIN_W(1) /BIN_E(6) |
|  |  |  | TZR1 | Slavkovský p. | ST | Dunajec a Poprad | 49.1315, 20.1988 | 1064 m | BIN_E(7) |
|  |  |  | POP3 | Poprad 3 | ST | Dunajec a Poprad | 49.1169, 20.0808 | 1224 m | BIN_E(1) |
|  |  |  | T37 | Tatranská Javorina | ST | Dunajec a Poprad | 49.1865, 20.1139 | 1339 m | BIN_E(1) |
|  |  |  | T177 | Gáborov p. | ST | Dunajec a Poprad | 49.1839, 19.8149 | 1408 m | BIN_E(2) |
|  |  |  | T36 | Podbanské | ST | Dunajec a Poprad | 49.1926, 20.0143 | 1497 m | BIN_E(1) |
|  |  |  | T023 | Temnosmreč 1 | ST | Dunajec a Poprad | 49.1945, 20.0300 | 1687 m | BIN_E(1) |
|  |  |  | T022 | Temnosmreč 2 | ST | Dunajec a Poprad | 49.1903, 20.0354 | 1713 m | BIN_E(1) |
|  |  |  | POP2 | Poprad 2 | ST | Dunajec a Poprad | 49.0837, 20.1420 | 817 m | BIN_E(1) |
|  |  | **FTA3**  Great Fatra (Veľká Fatra) | V033 | Jazierce | SP | Váh | 49.0182, 19.2819 | 589 m | BIN_W(1) /BIN_E(1) |
|  |  |  | MAT1 | Matejkovský p. | ST | Váh | 49.0010, 19.2631 | 728 m | BIN_W(1) |
|  |  | **FTA4**  Belianske Tatras (Belianske Tatry) | V036 | Dolina 7 prameňov | SP | Váh | 49.2228, 20.2776 | 1208 m | BIN_E(2) |
|  |  | **FTA5**  Strážov Mts (Strážovské vrchy) | NIT1 | Nitra 1 | ST | Váh | 48.6574, 18.6377 | 671 m | BIN_W(4) |
|  |  |  | TUR1 | Turiec 1 | ST | Váh | 48.9648, 18.7272 | 575 m | BIN_W(1) |
|  |  | **FTA6**  Choč Mts (Chočské vrchy) | V026 | Prosiek 1 | SP | Váh | 49.1621,19.4923 | 705 m | BIN_E(2) |
|  | **SCM**  Slovak Central Mts  (Slovenské stredohorie) | **SCM1**  Krupinská planina | KRI1 | Krivánsky | ST | Ipeľ | 48.4970, 19.5040 | 316 m | BIN_E(2) |
|  |  | **SCM2**  Poľana Mts (Poľana) | KAM1 | Kamenistý | ST | Hron | 48.6624, 19.6285 | 884 m | BIN_E(1) |
|  | **SOM**  Slovak Ore Mts  (Slovenské rudohorie) | **SOM1**  Slovak Karst (Slovenský kras) | V074 | Kunov Teplica | SP | Slaná | 48.6073, 20.3909 | 248 m | BIN_E(1) |
|  |  |  | V075 | Hučiaca B | SP | Slaná | 48.6252, 20.3899 | 269 m | BIN_E(4) |
|  |  |  | V088 | Drieňov kúpele | SP | Hornád a Bodva | 48.6245, 20.9520 | 257 m | BIN_E(2) |
|  |  | **SOM2**  Slovak Paradise (Slovenský raj) | V080 | Biele Vody | SP | Hornád a Bodva | 48.8741, 20.4041 | 850 m | BIN_E(1) |
|  |  |  | V082 | Pusté pole 2 | SP | Hornád a Bodva | 48.8840, 20.2041 | 953 m | BIN_E(2) |
|  |  |  | V084 | Mokrá dolina | SP | Hornád a Bodva | 48.8979, 20.2652 | 883 m | BIN_E(4) |
|  |  |  | V078 | bez mena 2 | SP | Hornád a Bodva | 48.8699, 20.2576 | 897 m | BIN_E(3) |
|  |  |  | V079 | Dobšinská ľad | SP | Hornád a Bodva | 48.8722, 20.3035 | 875 m | BIN_E(1) |
|  |  |  | V081 | Pusté pole 1 | SP | Hornád a Bodva | 48.8839, 20.2038 | 959 m | BIN_E(4) |
|  |  |  | V090 | Zejmarská | SP | Hornád a Bodva | 48.8776, 20.3972 | 1028 m | BIN_E(2) |
|  |  |  | VER1 | Hnilec 2 | ST | Hornád a Bodva | 48.8848, 20.2365 | 920 m | BIN_E(3) |
|  |  | **SOM3**  Muráň Plateau (Muránska planina) | RAC1 | Racov 1 | SP | Hron | 48.8520, 19.9959 | 659 m | BIN_E(1) |
|  |  | **SOM4**  Volovec Mts (Volovské vrchy) | KOJ1 | Kojšovský p. | ST | Hornád a Bodva | 48.8459, 21.0115 | 408 m | BIN_E(1) |
|  |  |  | SMO1 | Smolník 1 | ST | Hornád a Bodva | 48.7090, 20.7007 | 635 m | BIN_E(3) |
|  | **MSA**  Mátra-Slanec Area  (Severné stredohorie) | **MSA1**  Slanské Hills (Slanské vrchy) | HLB1 | Hlboký p. | ST | Hornád a Bodva | 48.9922, 21.4759 | 364 m | BIN_E(2) |
| **OWC** | **SMC**  Slovak-Moravian Carpathians  (Slovensko-moravské Karpaty) | **SMC1**  Javorníky Mts | VAH1 | Váh 1 | ST | Váh | 49.3253, 18.5111 | 582 m | BIN_W(2) |
|  | **WB**  Western Beskids  (Západné Beskydy) | **WB1**  Moravian-Silesian Beskids  (Moravsko-sliezske Beskydy) | CZ01 | Lomná | ST | Morava | 49.5477, 18.6504 | 538 m | BIN_W(3) |
|  |  |  | CZ02 | Příslopský | ST | Morava | 49.6242, 18.5754 | 497 m | BIN_W(1) |
|  |  |  | CZ03 | Satina | ST | Morava | 49.5653, 18.4228 | 772 m | BIN_W(8) |
|  |  |  | CZ05 | Kněhyně | ST | Morava | 49.4625, 18.2782 | 570 m | BIN_W(5) |
|  |  |  | CZ07 | Bystřička | ST | Morava | 49.3717, 17.7506 | 563 m | BIN_W(4) |
|  |  | **WB2**  Silesian Beskids  (Sliezske Beskydy) | PL04 | Żyłica | ST | Wisla | 49.6938, 18.9840 | 609 m | BIN_W(2) |
|  |  |  | PL05 | Labajów | ST | Wisla | 49.6228, 18.8692 | 523 m | BIN_W(9) |
|  | **PMA**  Podhale-Magura Area  (Podhôľno-magurská oblasť) | **PMA1**  Spiš Magura | HNC1 | Hraničná | ST | Dunajec a Poprad | 49.4168, 20.7232 | 386 m | BIN_E(3) |
|  |  |  | HRB1 | Hrebeniacky p. | ST | Dunajec a Poprad | 49.3358, 20.8432 | 437 m | BIN_E(2) |
|  |  |  | ZAV1 | Závoský p. | ST | Dunajec a Poprad | 49.3866, 20.7533 | 412 m | BIN_E(1) |
|  |  | **PMA2**  Oravská Magura | BRE1 | Brezovica | ST | Váh | 49.3438, 19.6621 | 687 m | BIN_E(5) |
| **IEC** | **VM**  Vihorlat Mts  (Vihorlatské vrchy) | **VM1**  Vihorlat Mts (Vihorlatské vrchy) | BAR1 | Barnov 1 | ST | Laborec | 48.9384, 22.1603 | 434 m | BIN_E(4) |
|  |  |  | HRA1 | Hrabový p. 1 | ST | Laborec | 48.8780, 22.2975 | 412 m | BIN_E(8) |
|  |  |  | JOV1 | Jovsiansky p. | ST | Laborec | 48.8265, 22.1049 | 176 m | BIN_E(2) |
|  |  |  | KRV1 | Krivec 1 | ST | Laborec | 48.9073, 22.2037 | 569 m | BIN_E(2) |
|  |  |  | LUH | Luhy 1 | ST | Laborec | 48.8598, 22.3424 | 304 m | BIN_E(1) |
|  |  |  | MOR1 | Morské oko | ST | Laborec | 48.9182, 22.1965 | 636 m | BIN_E(3) |
|  |  |  | SKA1 | Skalný p. | ST | Laborec | 48.8950, 22.2042 | 457 m | BIN_E(2) |
| **OEC** | **PM**  Poloniny Mts (Poloniny) | **PM1**  Poloniny Mts (Poloniny) | CIR1 | Cirocha 1 | ST | Laborec | 49.0228, 22.2518 | 282 m | BIN_E(3) |
|  |  |  | ULI1 | Ulička 1 | ST | Laborec | 49.0008, 22.3884 | 304 m | BIN_E(1) |
|  |  |  | PAC1 | Packov | ST | Laborec | 49.0510, 22.5147 | 418 m | BIN_E(1) |
|  | **LB**  Low Beskids (Nízke Beskydy) | **LB1**  Low Beskids (Nízke Beskydy) | HLP1 | Laborec | ST | Laborec | 49.3240, 21.9199 | 458 m | BIN_E(1) |

**Supplementary Table S3.** The list of all sampling sites and their physiographic affiliation. IWC – Inner Western Carpathians, OWC – Outer Western Carpathians, IEC – Inner Eastern Carpathians, OEC – Outer Eastern Carpathians; SP – spring, ST – stream; numbers in parentheses indicate the number of individuals for each locality.
